# Comment on ‘Adherence to Physical Activity and Incident Mobility Disability in Older Adults With Mobility Limitations’ by Alvarez‐Bustos et al.: The Authors' Reply

**DOI:** 10.1002/jcsm.70148

**Published:** 2025-12-02

**Authors:** Alejandro Álvarez‐Bustos, Emanuele Marzetti

**Affiliations:** ^1^ Department of Health Universidad Villanueva Madrid Spain; ^2^ Fondazione Policlinico Universitario Agostino Gemelli IRCCS Rome Italy; ^3^ Department of Geriatrics, Orthopedics and Rheumatology Università Cattolica del Sacro Cuore Rome Italy

We appreciate the authors' interest in our work on the relationship between adherence to physical activity interventions and the prevention of mobility disability. We are grateful for the critical insights they have provided, which highlight important methodological and clinical aspects that warrant consideration when assessing adherence and dose–response relationships.

The arguments raised by Zhang and Zhang [1] align with ongoing discussions in the field about refining trial designs to better capture the multifaceted and complex nature of physical activity interventions, particularly in older adults with mobility limitations. Regarding the potential confounding effect whereby participants with better baseline health or higher fitness levels may demonstrate greater adherence, we acknowledge this as a valid concern, since it could introduce selection bias into subgroup analyses. In our analyses, however, we attempted to account for key variables that could influence the results (i.e., age, sex, BMI, medical conditions, SPPB, MMSE, CES‐D and SARC‐F) [2], thereby reducing the impact of baseline subgroup traits. We also recognise that our study is inherently limited by the absence of other relevant factors (e.g., polypharmacy, personal motivation, fear of fatigue, social support and weather conditions) [3]. Future research exploring these and other variables will help optimise the prescription of physical activity for older adults and identify strategies to enhance adherence.

The suggestion to incorporate wearable sensors such as accelerometers, pedometers and heart rate monitors to establish comprehensive dose profiles is promising. However, in clinical practice and large multicentre trials, the use of such devices remains challenging. Indeed, the SPRINTT trial attempted to integrate a sensor‐based approach [4], but feasibility issues limited its implementation across all centres. Nevertheless, such tools may prove essential for exploring isotemporal substitution analyses, as proposed, and will hopefully be incorporated into future trials to better evaluate both supervised and unsupervised activity, thereby defining objective thresholds of frequency, intensity and type of exercise associated with mobility outcomes in this population.

The development of composite indexes that integrate attendance with intensity and duration is another interesting avenue. We agree that such scores, potentially weighted by moderate‐to‐vigorous physical activity, session bout length and perceived exertion, should be validated against clinical endpoints to ensure predictive value. It is important to note, however, that heart rate can provide biased estimates of resistance exercise intensity, particularly in older adults who are frequently prescribed medications (e.g., beta‐blockers, calcium channel blockers and anti‐arrhythmics) that alter cardiovascular responses. Moreover, in practical terms, monitoring these detailed parameters during group exercise sessions would be difficult for professionals to implement consistently. Thus, standardised methods that balance validity, feasibility and comparability across studies remain essential.

We would also like to thank Yang et al. [5] for their thoughtful scientific letter, which offers valuable perspectives on adherence monitoring and implementation strategies. As highlighted above, the use of real‐time monitoring through wearable sensors (e.g., ActiGraph and Fitbit) and AI‐driven platforms is consistent with the need for objective, affordable and scalable solutions to capture physical activity frequency, intensity and duration. We agree that integrating such technologies, particularly when coupled with user‐friendly interfaces, could allow individualised adherence goals tailored to participant characteristics, such as baseline SPPB scores. At the same time, the integration of these data into community‐based programmes, such as WHO's ICOPE framework, presents important challenges that must be addressed.

The proposal to leverage community health workers to sustain high adherence (> 3 sessions/week) in participants with SPPB 3–7 represents a practical and scalable strategy. We agree that community health workers trained in geriatric exercise protocols could improve adherence through home visits. However, in our study, visits were not conducted at participants' homes, and we did not differentiate between home‐based and centre‐based adherence, which could influence results. In settings where older adults cannot travel to exercise centres, incorporating supervised sessions via online platforms may provide an alternative means to support adherence.

Finally, we appreciate the recognition of broader policy‐level implications. We agree that expanding reimbursement beyond traditional programmes such as cardiac rehabilitation to include interventions targeting sarcopenia and frailty could incentivise participation. Likewise, infrastructure investments, such as safe walking paths, specialised gyms and dedicated training centres for older adults, could help reduce environmental barriers and support global ageing initiatives for populations with mobility limitations.

In summary, the thoughtful suggestions provided in both commentaries enrich the interpretation and generalisability of our findings while also pointing towards important avenues for future research, methodological refinement and clinical translation. Collectively, these insights reinforce the need for continued innovation in trial design, adherence monitoring and policy implementation to optimise physical activity interventions for older adults with mobility limitations.
